# Tumor microRNA profile and prognostic value for lymph node metastasis in oral squamous cell carcinoma patients

**DOI:** 10.18632/oncotarget.27616

**Published:** 2020-06-09

**Authors:** Kelly Yi Ping Liu, Sarah Yuqi Zhu, Denise Brooks, Reanne Bowlby, J. Scott Durham, Yussanne Ma, Richard A. Moore, Andrew J. Mungall, Steven Jones, Catherine F. Poh

**Affiliations:** ^1^Department of Oral Medical and Biological Sciences, Faculty of Dentistry, University of British Columbia, Vancouver, Canada; ^2^Department of Integrative Oncology, BC Cancer, Vancouver, Canada; ^3^Bioinformatics, Canada’s Michael Smith Genome Sciences Center, Vancouver, Canada; ^4^Department of Surgery, Faculty of Medicine, University of British Columbia, Vancouver, Canada; ^5^Faculty of Health Sciences, Simon Fraser University, Burnaby, Canada; ^6^Biospecimen & Library Core Group, Canada’s Michael Smith Genome Sciences Center, Vancouver, Canada

**Keywords:** oral squamous cell carcinoma, lymph node metastasis, micro-RNA, primary tumor, prognosis

## Abstract

Neck lymph node metastasis (LN+) is one of the most significant prognostic factors affecting 1-in-2 patients diagnosed with oral squamous cell carcinoma (OSCC). The different LN outcomes between clinico-pathologically similar primary tumors suggest underlying molecular signatures that could be associated with the risk of nodal disease development. MicroRNAs (miRNAs)are short non-coding molecules that regulate the expression of their target genes to maintain the balance of cellular processes. A plethora of evidence has indicated that aberrantly expressed miRNAs are involved in cancers with either an antitumor or oncogenic role. In this study, we characterized miRNA expression among OSCC fresh-frozen tumors with known outcomes of nodal disease (82 LN+, 76 LN0). We identified 49 differentially expressed miRNAs in tumors of the LN+ group. Using penalized lasso Cox regression, we identified a group of 10 miRNAs of which expression levels were highly associated with nodal-disease free survival. We further reported a 4-miRNA panel (miR-21-5p, miR-107, miR-1247-3p, and miR-181b-3p) with high accuracy in discriminating LN status, suggesting their potential application as prognostic biomarkers for nodal disease.

## INTRODUCTION

Worldwide, oral squamous cell carcinoma (OSCC) accounts for 274,000 new cases and 145,000 cancer-related deaths each year [1, 2]. Despite advances in treatment, the improvement of five-year survival rates (30–60%) is diminutive, mainly due to the proclivity of cancer cells to spread through the lymphatics system to neck lymph nodes, which reduces survival by half [3, 4]. Therefore, neck management has been part of the treatment planning for clinically node negative necks (LN0). Based on the premise that occult metastasis will inevitably progress into clinically manifest disease, a commonly practiced preventative strategy is elective neck dissection to remove the nodes at the time of surgical treatment. For some clinicians, this has started to become a part of the standard management plan for early-stage large size tumors (T3/4N0) or small tumors (T1/T2N0) with the depth of invasion (DOI) greater than 4 millimeters (mm). The association of DOI with biological behavior and tumor aggressiveness has been acknowledged in the latest edition of Cancer Staging Manual to incorporate DOI (cut-off of 5 mm) as the new additional staging criteria for OSCC [5]. Yet, this pathology is not definitive and the limited sensitivity and specificity have often been reported with the reminder that, not all large tumors will metastasize while a significant portion of small tumors does [6–8]. From our population-based retrospective study [9] and the pan-Canadian surgical trial, [10] 1-in-4 patients with clinically negative cervical nodal disease at the time of diagnosis (cLN0) develops LN+, inferring that only 25% of early-stage patients would benefit from END while others would receive unnecessary ND. Over-treating 75% of the patients incurs otherwise avoidable healthcare costs, potential complications, prolonged hospital stays, and morbidities. From a clinical management perspective, the decision whether to treat cLN0 patients is still controversial; therefore, searching for more sensitive and specific biomarkers that can stratify the risk of the nodal disease will offer more objective tool in nodal management, and consequently, better survival outcome.

The functions of microRNA (miRNAs) in biological processes, including cell growth, proliferation, and apoptosis, have led to reports of the integral parts in cancer progression through post-transcriptional modification of gene expression and/or translational repression [11, 12]. Alteration of miRNA expression can have a crucial role in cancer, and a growing body of evidence has shown that dysregulated miRNAs may be clinically meaningful with prognostic value [13]. MiRNAs with either oncogenic or tumor suppressor functions have also been highlighted in OSCC, with apparent differences between normal, pre-neoplastic lesions, and tumor tissues or cancer cell lines. The association of these markers with OSCC has been studied not only in primary tumors, but also biopsies, serum, and saliva, making them potential candidates for screening and diagnosis [14, 15]. To this end, however, only a few studies have focused on the prognostic value of miRNA in nodal disease [16–18]. In this study, we profiled the miRNA expression in primary OSCC tumors and generated a miRNA-based panel that could differentiate between LN-status groups.

## RESULTS

### Patient demographics and baseline tumor characteristics

The study population is summarized by Discovery and Validation cohorts in Table 1. Comparing between Discovery and Validation cohorts, there was no difference between LN+ and LN0 in age, sex, smoking history, primary tumor site, clinical T-stage prior to surgery, or tumor morphology except that LN+ had more tumors with greater depth of invasion (DOI) (*P* < 0.01).

**Table 1:** Patient demographics and baseline clinical-pathological characteristics

	Total	Discovery Cohort				Validation Cohort				
Variables	*N* = 158	Total (*n* = 91)	LN0 (*n* = 40)	LN+ (*n* = 51)	*P* (LN0 vs LN+)	Total (*n* = 67)	LN0 (*n* = 42)	LN+ (*n* = 25)	*P* (LN0 vs LN+)	*P* (Discovery vs Validation)
**Age, yrs (mean ± SD)**	62.6 ± 13.9	63.7 ± 14.6	62.15 ± 15.5	65 ± 14.0	0.31	61.5 ± 12.8	63.1 ± 12.9	58.7 ± 12.4	0.17	0.31
**Sex**					0.83				1	0.98
Male	98	57	26	31		41	26	15		
Female	60	34	14	20		26	16	10		
**Smoking History**									0.57	0.11
Never	68	35	16	20		32	20	12		
Ever	90	56	24	31		35	21	13		
**Primary tumor site**					1				1	0.94
Buccal mucosa/Gingiva/Hard Palate	16	9	3	6		7	7	5		
Soft Palate/Retromolar trigone/Soft Palate Complex	4	2	1	1		2	1	1		
Tongue/Floor of Mouth	138	80	36	44		58	36	22		
**Clinical T Stage**					0.69				0.14	0.93
T1/T2	148	85	38	47		63	41	22		
T3/T4	10	6	2	4		4	1	3		
**Clinical N Stage**					0.07				< 0.001	0.45
N0	138	83	40	43		56	42	13		
N+	20	9		9		11		11		
**Tumor Grade**					< 0.001				0.12	0.17
I	42	23	15	8		19	15	4		
II	78	42	22	20		36	22	14		
III	38	26	3	23		12	5	7		
**Tumor DOI (mean ± SD)**	7.0 ± 6.0	7.93 ± 6.78	5.7 ± 6.5	9.7 ± 6.5	< 0.01	5.81 ± 4.4			< 0.01	0.02
**Tumor DOI (4 mm)**					0.06				< 0.002	0.03
< 4 mm	4	21	13			27	24	3		
≥ 4 mm	110	70	27	43		40	18	22		
**Tumor DOI (5 mm)**					0.21				0.003	0.01
< 5 mm	62	29	16	24		33	27	15		
≥ 5 mm	96	62	13	38		34	6	19		
**Survival status**					< 0.001				0.002	0.04
Alive	105	54	35	18		51	37	14		
DOD	40	29	5	3		11	2	9		
Death (all causes)	13	8	0	29		5	3	2		
Time to death or last known date alive, y (mean ± SD)	3.2 ± 2.4	3.5 ± 2.4	4.9 ± 2.1	2.4 ± 2.0		2.9 ± 2.4	3.2 ± 2.6	2.6 ± 2.2	0.16	0.17

Abbreviation(s): DOI, depth of invasion; DOD, death of disease; LN0, lymph node negative; LN+, lymph node positive.

### miRNA expression clustering

**Figure 1:**
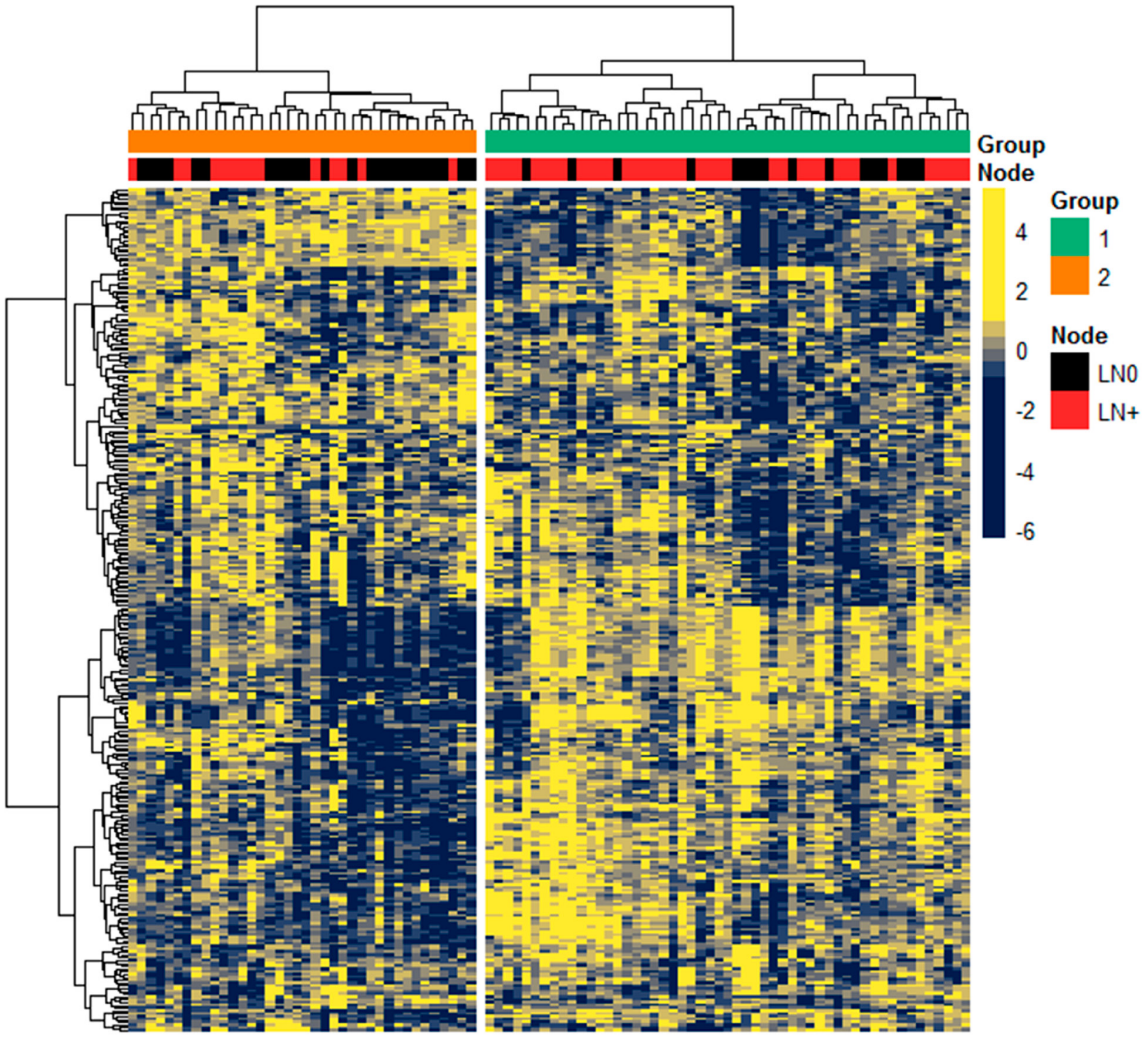
Expression of miRNA in Discovery cohort. Unsupervised hierarchical clustering of 301 miRNAs expression (scaled z-score) among the 91 patients, with Pearson correlation and Euclidean as distance measures for clustering the columns and rows, respectively. Top colored bars annotate the clustered groups derived from k-means clustering (*k* = 2) and LN status as either LN0 (black) or LN+ (red).

A total of 2075 miRNAs were annotated, of which 301 expressed at least 10 RPM in at least 10% of samples and were used for subsequent analysis. To determine the heterogeneity in miRNA expression, we performed unsupervised hierarchical clustering on the Discovery cohort (*n* = 91) with 2-group clustering (*k* = 2) based on our focus of comparing profiles between LN0 and LN+ status (Figure 1). The clustered groups (Group 1, *n* = 53; Group 2, *n* = 38) was significantly different in LN status (*P* = 0.0036, χ^2^), but not with other clinical-pathological variables, including 5-mm cut-off of DOI which is used to justify prophylactic neck dissection [19].

### Differentially expressed miRNAs between LN+ and LN0 tumors

**Figure 2:**
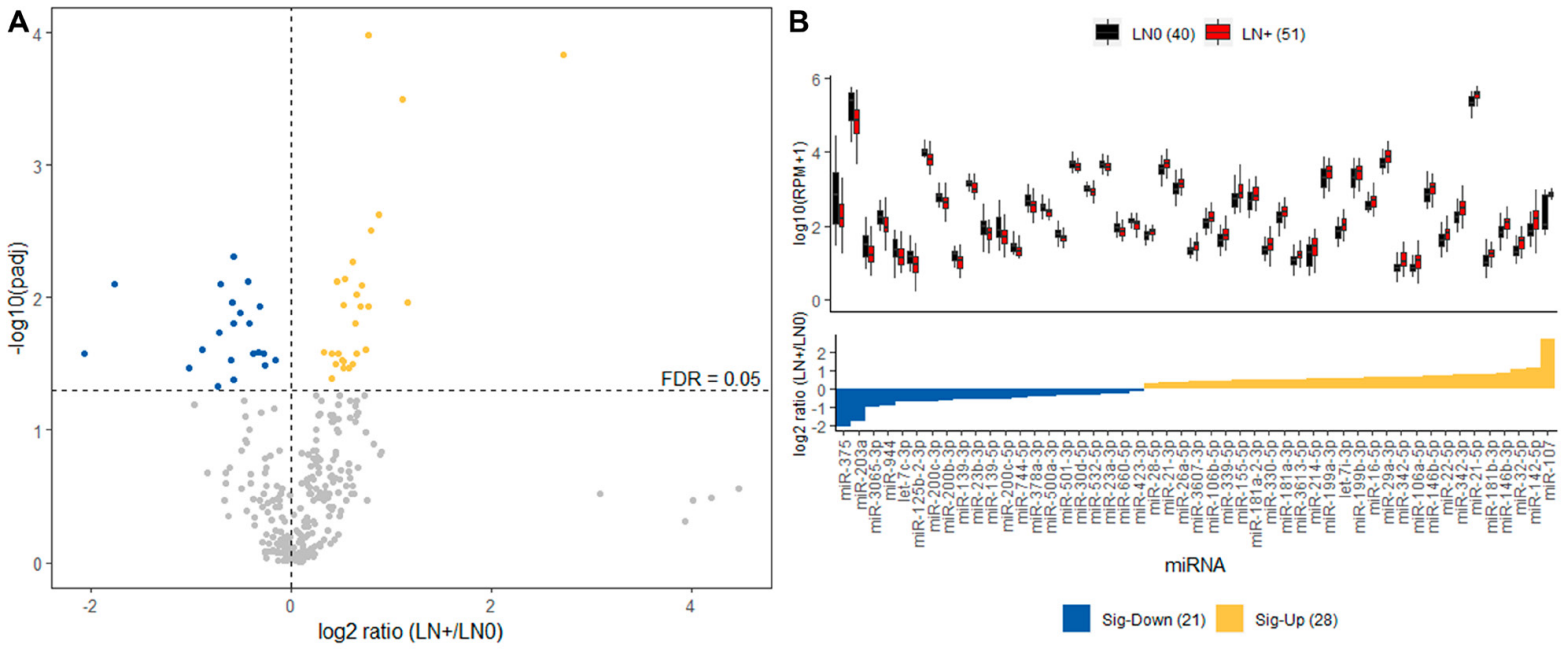
Differential expression analysis of Discovery cohort. (**A**) Volcano plot displaying differentially expressed miRNA between LN+ and LN0 groups. The 41 differentially up-regulated (yellow) and down-regulated (blue) miRNAs in LN+ group with FDR threshold of 0.05 (dashed line). (**B**) Top panel describes the distribution of expression for the 41 miRNAs in LN+ (red) and LN0 (black) group. Bottom panel displays the fold change for each of the 41 miRNAs where the yellow and blue indicates significantly up- and down-regulated in LN+, respectively.

In order to focus on differences in miRNA expression between LN+ and LN0, we performed DE analysis on the Discovery cohort using the Wilcoxon ranked-sum test for each miRNA. This revealed 49 (21 down- and 28 up-regulated) differentially expressed miRNAs as demonstrated by the fold change of LN+ against LN0 group after correction for multiple testing (Figure 2). As expected, several miRNAs that were significantly differentially expressed in LN+ were also among the miRNAs that contributed the most to the cluster separation, including most up-regulated miR-107 and down-regulated miR-375 (Table 2).

**Table 2:** Significantly differentially expressed miRNAs (FDR ≤ 0.05) in LN+ vs. LN0 primary tumors of the Discovery cohort

miRNA name and accession	*P*	FDR	Log2 FC
**Down-regulated**			
hsa-mir-375. MIMAT0000728	3.05E-03	0.026	–2.06
hsa-mir-203a. MIMAT0000264	3.15E-04	0.008	–1.76
hsa-mir-3065. MIMAT0015378	5.07E-03	0.034	–1.01
hsa-mir-944. MIMAT0004987	2.22E-03	0.025	–0.89
hsa-let-7c. MIMAT0026472	7.66E-03	0.047	–0.73
hsa-mir-125b-2. MIMAT0004603	1.52E-03	0.018	–0.71
hsa-mir-200c. MIMAT0000617	2.97E-04	0.008	–0.70
hsa-mir-200b. MIMAT0000318	3.65E-03	0.030	–0.60
hsa-mir-139. MIMAT0004552	5.77E-04	0.011	–0.59
hsa-mir-23b. MIMAT0000418	9.69E-05	0.005	–0.58
hsa-mir-139. MIMAT0000250	1.22E-03	0.016	–0.58
hsa-mir-200c. MIMAT0004657	6.63E-03	0.042	–0.57
hsa-mir-744. MIMAT0004945	9.18E-04	0.013	–0.51
hsa-mir-378a. MIMAT0000732	2.46E-04	0.008	–0.44
hsa-mir-500a. MIMAT0002871	1.15E-03	0.016	–0.42
hsa-mir-501. MIMAT0004774	2.90E-03	0.026	–0.37
hsa-mir-30d. MIMAT0000245	2.47E-03	0.026	–0.33
hsa-mir-532. MIMAT0002888	7.29E-04	0.012	–0.31
hsa-mir-23a. MIMAT0000078	2.90E-03	0.026	–0.28
hsa-mir-660. MIMAT0003338	4.59E-03	0.032	–0.27
hsa-mir-423. MIMAT0001340	3.75E-03	0.030	–0.15
**Up-regulated**			
hsa-mir-28. MIMAT0000085	2.41E-03	0.026	0.33
hsa-mir-21. MIMAT0004494	2.90E-03	0.026	0.41
hsa-mir-26a. MIMAT0000082	6.32E-03	0.040	0.41
hsa-mir-3607. MIMAT0017985	4.25E-03	0.032	0.45
hsa-mir-106b. MIMAT0000680	2.54E-04	0.008	0.45
hsa-mir-339. MIMAT0000764	2.90E-03	0.026	0.47
hsa-mir-155. MIMAT0000646	3.56E-03	0.030	0.51
hsa-mir-181a-2. MIMAT0004558	3.94E-03	0.030	0.52
hsa-mir-330. MIMAT0004693	5.20E-03	0.034	0.52
hsa-mir-181a-1. MIMAT0000270	6.49E-04	0.011	0.53
hsa-mir-3613. MIMAT0017990	1.91E-04	0.007	0.53
hsa-mir-214. MIMAT0004564	5.20E-03	0.034	0.58
hsa-mir-199a. MIMAT0000232	4.47E-03	0.032	0.62
hsa-let-7i. MIMAT0004585	1.26E-04	0.005	0.62
hsa-mir-199b. MIMAT0004563	4.36E-03	0.032	0.62
hsa-mir-16. MIMAT0000069	1.25E-03	0.016	0.64
hsa-mir-29a. MIMAT0000086	3.05E-03	0.026	0.65
hsa-mir-342. MIMAT0004694	4.41E-04	0.009	0.66
hsa-mir-106a. MIMAT0000103	7.73E-04	0.012	0.70
hsa-mir-146b. MIMAT0002809	3.46E-04	0.008	0.70
hsa-mir-22. MIMAT0004495	2.22E-03	0.025	0.75
hsa-mir-342. MIMAT0000753	7.08E-04	0.012	0.77
hsa-mir-21. MIMAT0000076	3.44E-07	0.000	0.78
hsa-mir-181b-1. MIMAT0022692	5.12E-05	0.003	0.80
hsa-mir-146b. MIMAT0004766	3.15E-05	0.002	0.88
hsa-mir-32. MIMAT0000090	3.20E-06	0.000	1.12
hsa-mir-142. MIMAT0000433	5.77E-04	0.011	1.17
hsa-mir-107. MIMAT0000104	9.70E-07	0.000	2.72

Abbreviation(s): FDR, false discovery rate; FC, fold change.

### MiRNAs associated with nodal-disease free survival

To investigate the association of these 301 miRNAs with time to nodal disease, we performed Cox proportional hazards (PH) analysis with patients categorized into low or high expression groups for each miRNA by determining the cut-point at which the *P* value of log-rank test is minimum (FDR threshold of 0.05). In the Discovery cohort (*n* = 91) (Supplementary Table 1), eight miRNAs showed significant difference in NFS between low and high groups (FDR < 0.05, hazard ratio (HR), 0.12 to 0.29/5 to 3.7e8). Subsequent multivariate analysis showed that these eight miRNAs were associated with NFS (*P* < 0.05; HR, 0.11 to 0.24/4.1 to 8.8e8), independently of cT stage (HR, 2.7 to 4.9), tumor grade (HR, 2.4 to 3.7), and DOI (HR, 1.0 to 1.1).

### miRNA-based prognostic models

**Figure 3:**
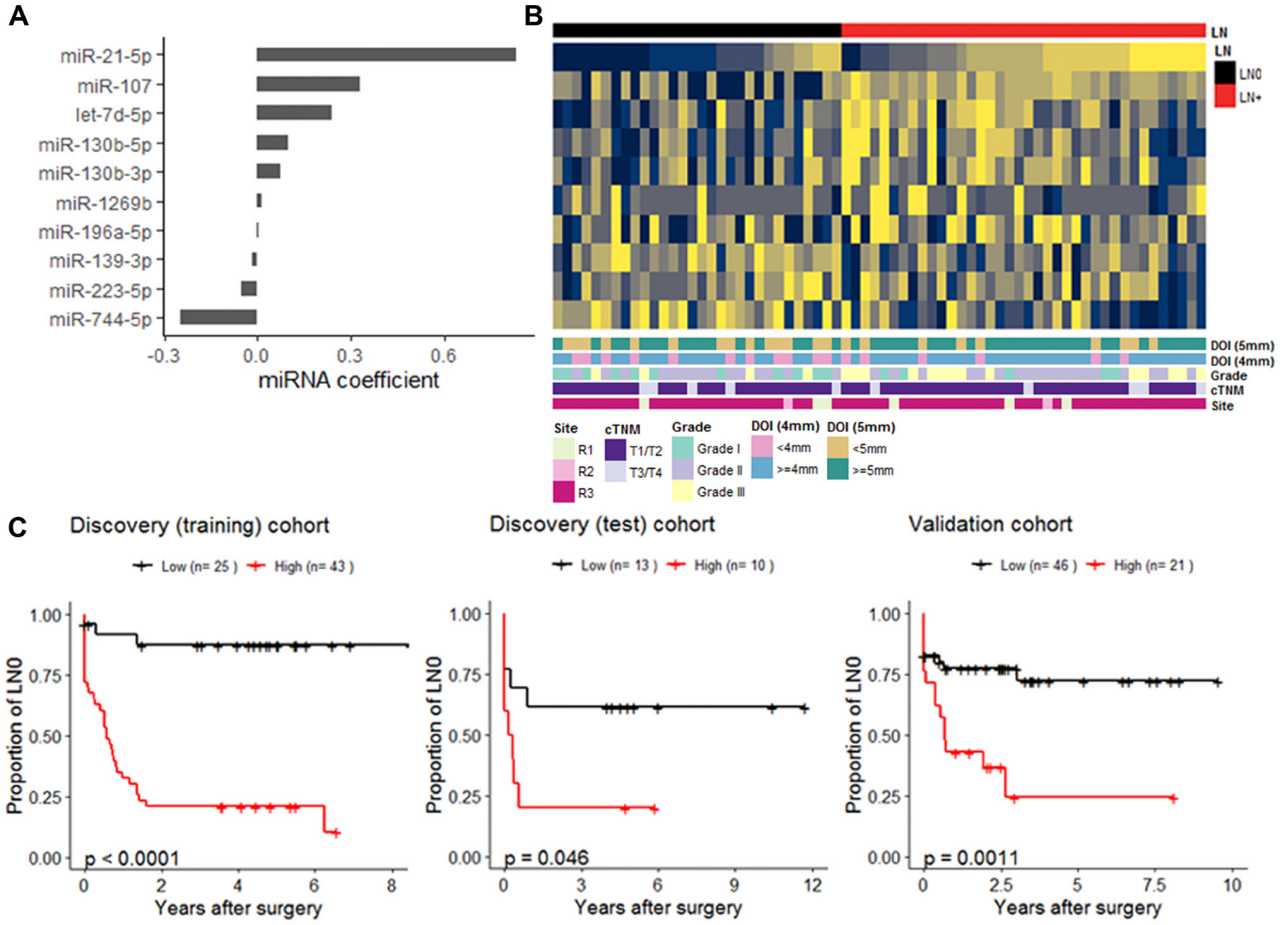
miRNA-based nodal-disease free (NFS) survival prognostic model. (**A**) Cox regression coefficients of the 10 miRNAs was generated from Discovery (training) cohort. (**B**) Heatmap of scaled expression of the 10 miRNAs in the Discovery (training) set which is annotated by the LN-status and other clinical-pathological attributes (**C**). Kaplan-Meier plots displaying NFS differences between patients in low and high groups within the Discovery (training) (*n* = 68), the Discovery (test) (*n* = 23), and the Validation cohort (*n* = 67). For each cohort, stratification of patients into low (black) and high (red) groups was determined by a cut-point of cox-model score based on the 10-miRNA coefficients (A) carried over from the Discovery (training).

The individual miRNAs identified by DE and Cox PH regression analyses suggest that multiple miRNAs had contributed to LN+. We explored this idea further using a penalized regression to generate a miRNA-based model as prognostic tool for NFS. For this penalized regression analysis, the Discovery cohort was randomly partitioned into a training set (*n* = 68; LN+, 37; LN0, 31) to generate the model, and a test set (*n* = 23; LN+, 14, LN0, 9) with no difference in the clinical-pathological characteristic (Supplementary Table 2). The Validation cohort was used as a second test set (*n* = 67; LN+, 25; LN0, 42). We used penalized lasso Cox PH regression on the Discovery (training) set to determine the regression coefficients for each miRNA. The resulting model included 10 miRNAs (Figure 3A and 3B), of which seven were overexpressed and three underexpressed in patients who experienced LN+. Using the coefficients of each of the 10 miRNAs generated from the model, we determined the cumulative score for each patient. The patients of each cohort were then separated into low or high score groups (Supplementary Table 2). By performing univariate Cox proportion hazard analysis, the high risk group had inferior outcomes in the Discovery (training) (HR = 11.3, 95% CI, 3.5–37.1; 5-year NFS, 20.9%, *P* < 0.001), Discovery (test) (HR = 2.9; 95% CI, 0.9–9.1.7; 5-year NFS, 20%, *P* = 0.046), and Validation cohort (HR = 2.4, 95% CI, 1.5–7.6; 5-year NFS, 24.5%, *P* < 0.001) (Figure 3C).

**Figure 4:**
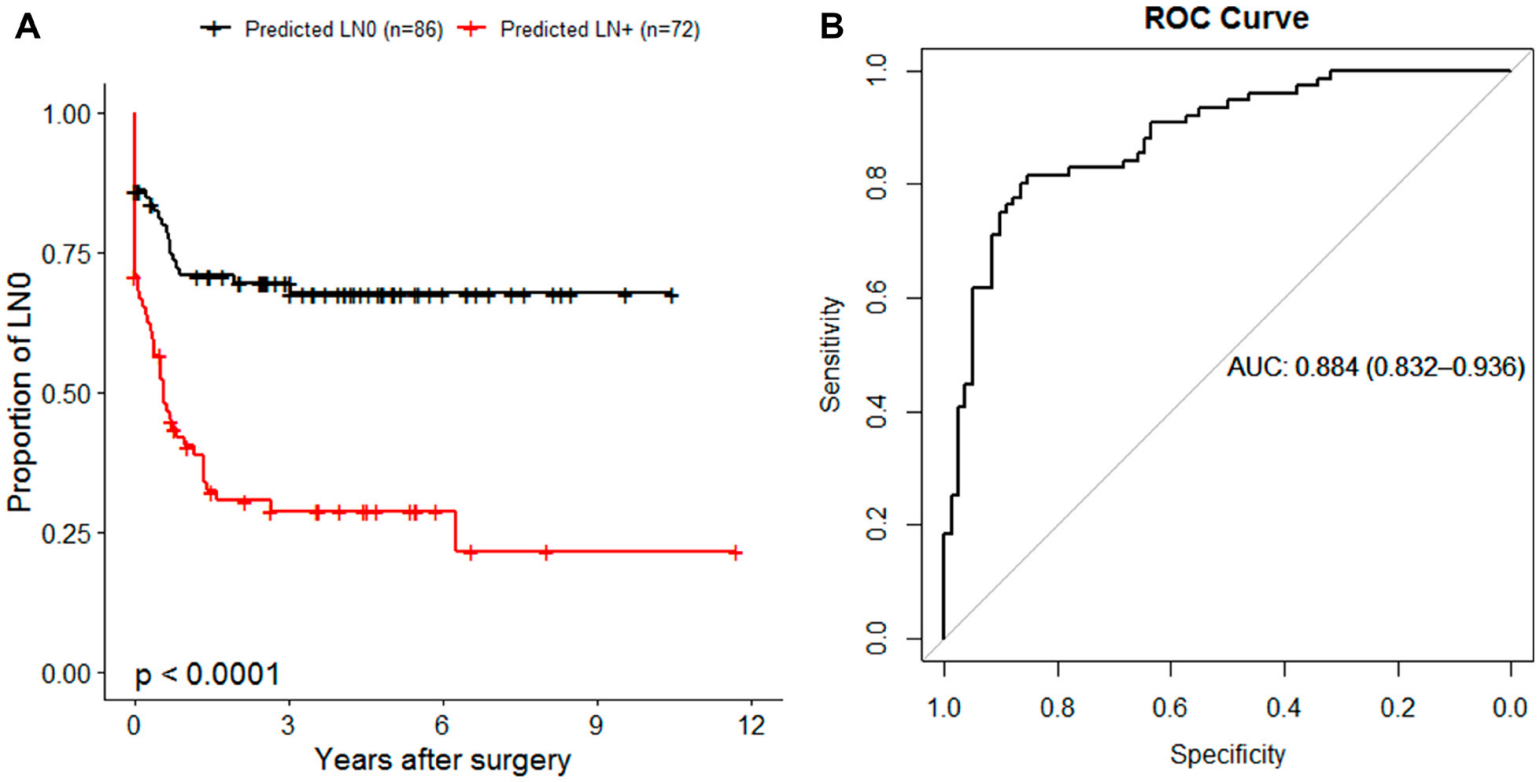
miRNA-based random forest (RF) classification of nodal disease status. (**A**) Kaplan-Meier plot illustrating the NFS of 158 patients in RF predicted groups of LN0 (black) or LN+ (red). (**B**) Receiver Operator Characteristic (ROC) curve generated by plotting the 4-miRNA classifier true positive rate (sensitivity) as function of the false positive rate (1-specificity). The RF prediction probabilities were used for the generation of ROC prediction objects. The area under the curve (AUC) is reported as a performance measure.

To identify potential miRNAs that can be used to indicate binary outcome of LN, we implemented random forest classification approach on the entire study population (*n* = 158). We first performed miRNA selection based on the variable importance by recursive eliminating those with the smallest importance. This yielded 146 miRNAs that were used to generate the model which correctly separated LN status for 122 out of 158 samples, showing strong correlation with NFS (*P* < 0.001) (Figure 4A). To identify the miRNAs that could have potential clinical value, we performed a step-wise approach based on the 146 miRNAs with smallest out-of-bag error rate. The fitted model consists of four miRNAs (miR-107, miR-21-5p, miR-1247-3p, miR-181b-3p), which were consistent with penalized Cox PH regression analysis with performance of AUC = 0.88 in discriminating LN status (Figure 4B).

## DISCUSSION

Metastatic lymph node in the neck is the first indication of tumor spread in OSCC patients and one of the most significant prognostic factors. For patients with occult metastasis, clinically applicable biomarkers may be useful in clinical decision making on the management of cN0 neck and improve survival. MiRNAs are small non-coding RNA molecules (each containing ~22 nucleotides) known to be preserved in archival formalin-fixed paraffin-embedded tissues, making them desirable tools to be used as potential biomarkers. In the present study, we identified dysregulated miRNAs of primary OSCC tumors associated with nodal disease.

Several differentially expressed miRNAs in our datasets are commonly associated with OSCC progression and prognosis, namely miR-21, miR-107. Both of these also contributed to both the Cox-PH prognostic model and random forest classification model, suggesting that they can be used as potential biomarkers to identify patients with a high risk of nodal disease. MiR-21 is commonly considered as an oncogene in solid tumors, and its up-regulation has been widely associated with prognostic value [20]. Its correlation with the presence, progression, or invasiveness of OSCC has been demonstrated in not only cell lines and tissue samples, but also blood samples, encouraging its prognostic and clinical value [21–23]. For example, using *in-situ* hybridization on archival surgical tissues from OSCC patients, Hedback *et al*. correlated miR-21 expression in both tumor stroma and tumor cells with disease-specific survival in OSCC patients [24]. More recently, Yu *et al.* reported association of upregulation of miR-21 with patient survival independently from clinical-pathological factors, including tumor size, clinical stage, and lymphovascular or perineural invasion [25]. A few mechanisms of this miRNA in oncogenic events have been proposed, including epithelial-mesenchymal transition by targeting phosphatase and tensin homolog (PTEN), angiogenesis and metastasis in liver and lung; [26–28] or by promoting tumor cell migration through regulating metalloproteinase inhibitor 3 (TIMP-3) transcription and promoting migration in cervical cancer [29]. In addition, increased expression of miR-21 was also associated with tumors characterized by p53 mutations and distant metastasis [30].

Another significantly up-regulated miRNA observed in LN+ tumors is miR-107, a highly conserved miRNA that contributes to the regulation of normal and tumor biological processes, including cell division, metabolism, and angiogenesis [31, 32]. Dysregulation of miR-107 in human tumors has been significantly associated with disease staging, metastasis, and treatment outcomes; [31, 33] however, the oncogenic or antitumor role of miR-107 has been debated in studies of head and neck cancer. For instance, miR-107 was found to be highly expressed in tongue cancer cell lines, while a later study showed that it was downregulated in OSCC cell lines and tongue SCC tissues [34, 35]. In a study involving head and neck cell lines, including tongue SCC, Datta *et al.* suggested a therapeutic role of miR-107 as they observed an inverse relationship between expression of miR-107 and *PRKCE* gene, protein kinase C (PKC) epsilon. This gene is often reported to be elevated in head and neck cancer involving signal transduction pathways of proliferation and migration [36]. Another recent study unraveled the tumor-suppressor role of miR-107 in esophageal cancer by targeting *CDC42* [37]. From what we can gather, the role of miR-107 is still poorly understood, with few investigations on the association with lymph node metastasis.

Ideally, a clinically applicable biomarker should be able to stratify at-risk patients of progression early enough in the course of treatment to consider elective neck dissection, namely precision medicine. Here, we identified a 4-miRNA-based (miR-21-5p, miR-107, miR-181b-3p, miR-1247-3p) prognostic model that was able to stratify patients by LN status with high accuracy. Of these, miR-21-5p and miR-107 were also significantly correlated with nodal-disease free survival, where the model-based high-risk patients had inferior survival. Up-regulation of miR-181 has been reported in OSCC transformation from leukoplakia, dysplasia to invasive tumor. In a study surveying expression of miR-181 family in OSCC tissue samples and blood plasma, Yang *et al.* reported the association of miR-181b up-regulation with lymph node metastasis and vascular invasion, which was supported by the observed enhanced cell migration in miR-181 transfected cell lines in the same study [38]. Although the role of miR-181 in metastasis is still not well understood, the discriminative performance observed in our study warrants further investigation studies to validate the clinical significance of the up-regulation of miR-181 in nodal disease. Although the expression of miR-1247 was not significantly different between clinical subgroups, it was retained as one of the predictors powering separation of our dataset into LN0 and LN+ groups. Although there is little research on the role of miR-1247 in OSCC, Fang et al. reported that miR-1247 is highly elevated in metastatic liver cancer cells and cancer-cells-derived exosomes that act as a mediator in the activation of cancer-associated fibroblasts, leading to tumor progression and metastasis [39]. In the same study, the expression of miR-1247 was also observed with increased pro-inflammatory gene expression, such as IL6 and IL8. Given that OSCC is often characterized by heavily infiltrated inflammatory cells, perhaps future in-depth studies on the association between miR-1247 and tumor microenvironment may explain the observed association with nodal disease in this study.

To assess the possible combinatorial effect of the four miRNAs, the Kyto Encyclopedia of Genes and Genomes (KEGG) pathway analysis was performed by using mirPath tool (v3.40) [40]. Significantly enriched pathways (Fisher’s exact test *P* values < 0.05) in the LN+ group involving the four miRNAs include the Hippo signaling pathway and signaling pathways regulating the pluripotency of stem cells. Given the metastatic nature of these cells, it is not surprising the inhibition of these pathways in order to maintain the initiation and maintenance of tumorigenicity [41]. In addition, these miRNAs are known to be involved in tumor growth and progression in many cancer types, [13] which is reflected with colorectal cancer and pathways in cancer among the most regulated pathways. The most regulated pathway is fatty acid (FA) biosynthesis and metabolism involving miR-107 targeting fatty acid synthase (*FASN*). Hyperproliferating cancer cells require FA for energy metabolism and storage, membrane building block synthesis, and singling molecules synthesis. Therefore, disrupting FA metabolism neglects the high metabolic demands of cancer cells which could suppress tumor growth and metastatic dissemination. On the contrary, we observed an overexpression of miR-107 in the LN+ group which suggested that nodal metastasis may be promoted with decreased activity of lipogenesis. We speculate that with high metabolic demands and under hypoxic environment, aggressively metastatic cancer cells excessively upregulate their FA synthesis and uptake that can lead to FA accumulation and lipotoxicity, and ultimately, cell death [42]. miR-181b is involved in a few cellular processes together with miR-107. One of them is lysine degradation. Given that amino acid metabolism is a major source of energy and carbon for tumor cell growth and survival, the dysregulation of lysine degradation pathway may result from changes in the energy metabolism of tumor cells.

We observed several miRNAs that are significantly differentially expressed between the LN status groups, yet they do not effectively contribute to the Cox-PH or RF classification models. This leads to speculation that there are other clinical-pathological factors or the tumor microenvironment driving the differences. One of these is miR-375 which was the most down-regulated miRNA in LN+ tumors in this study. The down-regulation of miR-375 has been reported elsewhere in OSCC comparing tumor to adjacent normal tissues, and in metastatic cancer cell lines [43–45].

This study has limitations. First, validation of miRNA biomarkers on RT-PCR was not performed. This study, as the first step, was to explore and identify significantly dysregulated miRNAs in LN+ for which we used an independent set of samples to verify the observed miRNA expression in the Discovery cohort. Second, as this is not a biological or functional study, mRNA-miRNA interaction and target gene analyses were not performed. However, this study indicates future directions, including validation of the observed expression and prognostic power on RT-PCR, NanoString, or RNAScope *in situ* hybridization. Target gene analysis and investigation on the biological functions of these miRNAs may help us to understand the underlying mechanism of lymph node metastasis. The goal is the clinical applications of these biomarkers on small biopsied FFPE samples to decide the need for neck treatment before surgery. Hence, the prognostic power will need to be verified on FFPE surgical samples and small biopsy samples for pre-surgery planning. Alternatively, a fresh-frozen re-biopsy at tertiary center prior of surgery can also be considered.

OSCC is a heterogeneous disease, and metastasis is most likely to be attributed to many factors. In this study, we performed miRNA profiling on tumor samples and identified dysregulated miRNAs, among of which four miRNAs were observed with high performance to discriminate LN status. These biomarkers may provide additional information and may be able to identify patients with a low chance of nodal disease. However, sequencing miRNA expression from surgically removed tumors may limit these miRNAs’ clinical relevancy as studying bulk tumors neglects the reality that diagnostic biopsy examination is used for treatment planning. This concern warrants future investigations to verify the observed prognostic power in not only matched archival FFPE surgery samples but also in diagnostic biopsy material on clinically adaptable platforms to support their clinical relevance in neck management for early-stage OSCC.

## MATERIALS AND METHODS

### Patient and tissue collection

Patients diagnosed with OSCC who received primary surgical excision between 2005 and 2016 were identified through a longitudinal surgical trial. Of the 487 patients enrolled, we were able to retrieve 158 primary tumors collected at time of surgery that were embedded in OCT compound and frozen immediately after excision in –80°C until use. Table 1 summarizes baseline demographics and clinical-pathological data. Outcome data included binary status of LN0 or LN+, and nodal-disease free survival (NFS), which was measured from date of surgery to date of metastatic OSCC in the cervical lymph nodes. Patients who were last known to be alive and nodal-disease free were censored at the date of last contact. Samples were randomly submitted into two batches for sequencing as Discovery and Validation cohorts. HPV status was determined on 89 samples with enough DNA material by using multiplex PCR with high-risk HPV-16 and HPV-18 primers. Of these, only one patient was positive for HPV-16; therefore, we did not perform comparative analysis between groups on HPV.

The study was conducted in accordance with the recommendation of University of British Columbia Clinical Research Ethics and the BC Cancer Research Ethics General Guidance Notes, BC Cancer Research Ethics Board. This study utilized the clinical information and samples collected from existing studies approved by the BC Cancer Research and Ethics Board (REB# H09-03090 and REB#17-02031).

### RNA extraction and miRNA library preparation

Total RNA was harvested from the fresh-frozen samples, which were collected in the operating room at the time of surgery, by either taking tissue cores or microdissection from areas containing at least 70% tumor content after being reviewed by an oral pathologist (CFP). Nucleic acid extraction was performed using AllPrep DNA/ RNA/miRNA Universal kits (QIAGEN, CA, USA) as per the manufacturer’s protocol. The RNA concentration was determined by NanoDrop (Thermo Scientific, CA, USA). The RNA submitted for sequencing had a concentration of 100 ng/μL and RNA integrity number (RIN) ≥8 as determined by the Bioanalyzer 2100 RNA 6000 Nano Kit (Agilent Technologies, CA, USA).

Small RNAs 20–30 nucleotides in length, including microRNAs (miRNAs), were captured from total RNA or total nucleic acids extracted from tissues using a protocol implemented at Canada’s Michael Smith Genome Sciences Centre on Microlab NIMBUS (Hamilton) liquid handlers. Briefly, 500 ng of total RNA in 8 μL diethyl pyrocarbonate (DEPC)-treated water was first ligated to 2 μL of a 2.5 μM 3′ DNA adapter in a 96-well microtitre plate followed by incubation (70°C for 2 min), snap chilled, and transferred to 10 μL ligation brew containing truncated T4 RNA ligase 2 (200 U/μL, New England Biolabs). Excess adapter was removed by incubation (1 hour at 22°C) and purification twice using RNA MagClean DX beads (Aline Biosciences). The 3′ adapter-ligated RNAs were next ligated to heat denatured 5′ miRNA adapter using T4 RNA ligase (5 U/μL, Ambion) followed by incubation (1 hour at 37°C) and reverse transcription with Maxima H minus reverse transcriptase (RT) primer (200 U/μL) by incubation (10 minutes at 65°C) and snap chilled on ice. First strand cDNA was purified using an upper and lower bead clean-up (PCRClean DX beads, Aline Biosciences) to remove excess RT primer and reduce non-target products prior to PCR enrichment. PCR (15 cycles) was performed using a paired-end primer and miRNA indexed primers in a 50 μL reaction volume incorporating Phusion Hot Start high fidelity DNA polymerase (NEB). The amplified library was loaded onto a 12% PAGE gel and the region containing the miRNA library (~150 bp) was manually excised from the gel (size-selected). The size-selected library was ethanol precipitated and purified. Quality control of the final library was performed using Qubit and Agilent DNA 1000 Series II assays prior to sequencing on an Illumina NextSeq500 instrument generating single-end 75 base reads. The sequenced miRNA data from 158 samples were aligned to NCBI GRCh37/hg19 and annotated based on mirBase (version 20) 5p or 3p mature strands, a repository of previously annotated miRNAs.

### Statistical analysis

Patient baseline demographics and clinical-pathological characteristics were described as continuous variable (mean ± SD) or categorical variables in frequency (n) and proportion (%). Chi-square test was used for proportion comparison while Student’s *t*-test for difference in distribution. All statistical tests at *P* ≤ 0.05 were considered significant. Statistical analysis was performed using the software R (3.4.4) packages.

Analysis of expression profiles was performed on annotated miRNAs with largest variances (top 90%, *n* = 301) across the Discovery cohort after normalization and removal of miRNAs with low number of reads (<10 reads-per-million (RPM) in less than 10% of the samples). To identify subtypes within the cohort we used hierarchical clustering with input of log10 transformed data matrix. We used ward. D2 for the clustering method with Pearson correlation and Euclidean as the distance measures for the clustering of the columns and rows, respectively. Evaluation of the differential expression (DE) miRNA between subgroups was performed using the Wilcoxon ranked-sum test for each miRNA. Significantly differentially expressed miRNA had Benjamini-Hchoberg (BH) multiple test corrected *P* values (FDR) ≤ 0.05.

Association between miRNA expression and nodal-disease free survival (NFS) was first investigated on the Discovery cohort by using Cox proportional hazards (PH) regression analysis. We first determined the cut-point for each miRNA (log10 RPM) at which could separate the patients into low/high groups with minimum *P* value of log-rank test. miRNAs that were significant after correction for multiple testing (FDR ≤ 0.05 at determining cut-point and across all miRNAs) were subjected to univariate and multivariate Cox-PH analysis with clinical T stage, tumor grade, and tumor DOI.

To generate miRNA-based prognostic model, we performed penalized Cox regression (glmnet v2.0-16) on a randomly partitioned subset of Discovery (training) cohort. The Cox regression derived regression coefficients from Discovery (training) were carried over to Discovery (test) and the Validation cohort to establish a score for prognosis risk of NFS based on linear combination of miRNA expression multiplied by the miRNA coefficient. Based on the risk score, we then determined the cut-point that stratified patients into low/high risk groups based on 5-year NFS with smallest Kaplan-Meier log-rank *P* value (maxstat v0.7-25). Finally, random forest classification analysis (randomForest v4.6) was performed on the entire study population to build predictive model (1000 trees, each using 9 miRNAs as predictors) for LN status with variable selection based on out-of-bag (OOB) error rate (VSURF 1.1.0). We further select the number of variables for prediction by sequentially introducing variables until the decrease in error rate is negligible. The final miRNA panel was analyzed with receiver operating curve (ROC) analysis to test the performance of discriminating LN status [40].

## SUPPLEMENTARY MATERIALS


