# Supplementary material for: Tumor microRNA profile and prognostic value for lymph node metastasis in oral squamous cell carcinoma patients
Source: Oncotarget. 2020 Jun 9;11(23):2204–15. doi: 10.18632/oncotarget.27616 (PMC7289532; doi:10.18632/oncotarget.27616)
Supplement: Supplementary file 1 [file oncotarget-11-2204-s001.pdf]

Tumor microRNA profile and prognostic value for lymph node metastasis in oral squamous cell carcinoma patients

SUPPLEMENTARY MATERIALS

Supplementary Table 1: miRNAs significantly associated with nodal disease-free survival by cox-proportional hazard analysis on low or high miRNA expression

				Univariate		Multivariate with cT stage, tumor grade, DOI	
miRNA name and accession	cut-point (log10 RPM)	FDR[a]	Log-rank *P*[b]	HR (95% CI)	Log-rank *P*	HR (95% CI)	Log-rank *P*
hsa.mir.106a.MIMAT0000103	3.102	0.03711	0.00098633	3.4 (1.9–6)	2.60E-05	2.3 (1.2–4.4)	1.00E-13
hsa.mir.107.MIMAT0000104	6.802	0.00005	0.00000015	2.7 (1.6–4.4)	1.40E-09	8.8e8 (0–Inf)	9.00E-16
hsa.mir.146b.MIMAT0004766	6.224	0.00764	0.00010158	1.9 (1.4–2.7)	7.80E-05	4.1 (1.8–9.5)	7.00E-14
hsa.mir.181b.1.MIMAT0022692	3.264	0.00082	0.00000821	1.7 (1.3–2.3)	2.50E-07	7.2 (2.5–21.3)	2.00E-14
hsa.mir.200c.MIMAT0000617	12.974	0.02905	0.00067568	0.29 (0.15–0.53)	2.60E-05	0.24 (0.13–0.47)	6.00E-16
hsa.mir.203a.MIMAT0000264	17.879	0.00778	0.00014545	0.12 (0.038–0.39)	1.60E-06	0.11 (0.034–0.39)	1.00E-14
hsa.mir.21.MIMAT0000076	17.859	0.00011	0.00000073	8 (3.2–20)	1.90E-08	8.3 (3.2–22)	6.00E-16
hsa.mir.32.MIMAT0000090	4.159	0.00778	0.00015514	8.2 (2.5–26)	1.60E-06	8.7 (2.6–29)	3.00E-15

[a]*P* values were corrected for multiple testing at determining cut-point at false discovery rate (FDR) threshold of 0.05.
[b]*P* values were corrected for multiple testing at determining association with nodal-disease free survival across 301 miRNAs.
Abbreviation(s): RPM, reads per million; HR, hazard ratio; CI, confidence intervals.

Supplementary Table 2: Patient and baseline clinical-pathological characteristics between low and high score groups in Discovery (training), Discovery (test), and validation cohorts

	miRNA score group based on Penalized Cox PH regression model											
	**Discovery (training) cohort**	**Discovery (test) cohort**		**Discovery (training) cohort**			**Discovery (test) cohort**			**Validation cohort**		
	**(*n* = 68)**	**(*n* = 23)**	***P***	**Low (25)**	**High (43)**	***P***	**Low (13)**	**High (10)**	***P***	**Low (46)**	**High (21)**	***P***
**Age, yrs (mean ± SD)**	63.7 ± 14.4	63.6 ± 15.8	0.98	66.7 ± 13.6	62.0 ± 14.7	0.18	58.2 ± 15.8	70.7 ± 13.3	0.05	63.1 ± 13.1	58.0 ± 11.7	0.12
**Sex**			1			0.16			0.61			1
Male	43 (63)	14 (61)		19 (76)	24 (56)		4 (31)	5 (50)		18 (39)	8 (38)	
Female	25 (37)	9 (39)		6 (24)	19 (44)		9 (69)	5 (50)		28 (61)	13 (62)	
**Smoking History**			0.92			0.73			0.56			0.41
Never	27 (40)	9 (39)		11 (44)	16 (37)		5 (38)	4 (40)		20 (43)	12 (57)	
Ever	41 (60)	14 (61)		14 (56)	27 (63)		8 (62)	6 (60)		26 (57)	9 (43)	
**Primary tumor site**			0.83			0.53			1			0.61
Buccal mucosa/Gingiva/ Hard Palate	6 (9)	3 (13)		1 (4)	5 (12)		2 (15)	1 (10)		5 (11)	2 (10)	
Soft Palate/Retromolar trigone/Soft Palate Complex	2 (3)	0 (0)		1 (4)	1 (2)					2 (4)	0 (0)	
Tongue/Floor of Mouth	60 (88)	20 (87)		23 (92)	37 (86)		11 (85)	9 (90)		39 (85)	19 (90)	
**Clinical T Stage**			0.75			0.82			0.16			0.54
T1/T2	58 (85)	19 (83)		21 (84)	37 (86)		12 (92)	7 (70)		38 (83)	16 (76)	
T3/T4	10 (15)	4 (17)		4 (16)	6 (14)		1 (8)	3 (30)		8 (17)	5 (24)	
**Clinical N Stage**			0.56			0.86			0.38			0.69
N0	62 (91)	20 (87)		23 (92)	39 (91)		12 (92)	8 (80)		39 (85)	17 (81)	
N+	6 (9)	3 (13)		2 (8)	4 (9)		1 (8)	2 (20)		7 (15)	4 (19)	
**Tumor Grade**			0.62						0.98			0.06
I/II	50 (74)	15 (65)		8 (32)	8 (19)		9 (69)	6 (60)		41 (89)	14 (67)	
III	18 (26)	8 (35)		14 (56)	20 (47)		4 (31)	4 (40)		5 (11)	7 (33)	
**Tumor DOI (mean ± SD)**	7.97 ± 7.24	7.83 ± 5.30	0.92	6.3 ± 8.2	8.9 ± 6.6	0.19	6.3 ± 3.9	9.9 ± 6.3	0.14	4.7 ± 3.5	8.3 ± 5.1	0.005
**Tumor DOI (4 mm cut-off)**			0.64			0.19			0.79			0.008
< 4 mm	17 (25)	4 (17)		9 (36)	8 (19)		3 (23)	1 (10)		24 (52)	3 (14)	
≥ 4 mm	51 (75)	19 (83)		16 (64)	35 (81)		10 (77)	9 (90)		22 (48)	18 (86)	
**Tumor DOI (5 mm cut-off)**			0.67			0.11			1			0.04
< 5 mm	23 (34)	6 (26)		12 (48)	11 (26)		3 (23)	3 (30)		27 (59)	6 (29)	
≥ 5 mm	45 (66)	17 (74)		13 (52)	32 (74)		10 (77)	7 (70)		19 (41)	15 (71)	

Abbreviation(s): DOI, depth of invasion.
